# Gilteritinib Combined with Azacitidine as Salvage Therapy for B/Myeloid Mixed Phenotype Acute Leukemia

**DOI:** 10.7759/cureus.23618

**Published:** 2022-03-29

**Authors:** Nathan P Horvat, Constantine N Logothetis, Ling Zhang, Seongseok Yun, Kendra Sweet

**Affiliations:** 1 Oncology, University of South Florida Morsani College of Medicine, Tampa, USA; 2 Internal Medicine, University of South Florida Morsani College of Medicine, Tampa, USA; 3 Hematopathology, Moffitt Cancer Center, Tampa, USA; 4 Malignant Hematology, Moffitt Cancer Center, Tampa, USA

**Keywords:** fms-like tyrosine kinase 3, azacitidine, gilteritinib, biphenotypic acute leukemia, mixed phenotype acute leukemia

## Abstract

Mixed phenotype acute leukemia (MPAL) is a rare group of acute leukemias with blasts that co-express antigens of more than one lineage or separate populations of blasts of different lineages. Though treatment guidelines are not well established, the standard of care in treating MPAL remains the acute lymphoblastic leukemia (ALL)-derived chemotherapeutic regimen of hyper-cyclophosphamide, vincristine, doxorubicin (also known by its trade name, Adriamycin), and dexamethasone (CVAD) followed by allogeneic stem-cell transplant (ASCT). Beyond induction chemotherapy, evidence-based treatments remain to be investigated, especially regarding patients who relapse prior to ASCT. This case report illustrates a patient with relapsed MPAL following induction hyper-CVAD who was not immediately eligible for ASCT. After brief treatment with gilteritinib alone, the patient was started on gilteritinib and azacitidine as salvage therapy and achieved and maintained complete remission with incomplete count recovery (CRi) for eight months. Targeted therapy is a novel approach to improve survival rate, but unfortunately, there have been very few studies in the context of MPAL. We report a patient with relapsed FLT3-mutant MPAL who achieved remission using a combination approach with targeted therapy.

## Introduction

Mixed phenotype acute leukemia (MPAL), previously referred to as biphenotypic acute leukemia, is defined by the World Health Organization (WHO) as a group of leukemias with blasts that co-express antigens of more than one lineage or separate populations of blasts of different lineages [1]. Regardless of the presence of more than one population, MPAL is further characterized as T/myeloid, B/myeloid, or rare types (e.g., T/B phenotypes) based on the antigens expressed by the blasts, with or without genetic aberrations involving t(9;22)/BCR-ABL1 or t(r:11q23.3)/KMT2A rearrangements. MPAL patients can present with signs of bone marrow failure such as fatigue, infections, bleeding, and an elevated white blood cell count and high number of circulating blasts [2].

The incidence of MPAL is much lower compared to acute myeloid leukemia (AML) and acute lymphoblastic leukemia (ALL), and it has a bimodal age distribution, with the peak incidences in people younger than 19 years and older than 60 years [3]. Compared to AML and ALL, MPAL has a decreased overall survival (OS) and a poor prognosis for achieving complete remission (CR) [4-6], although some more recent studies have shown that MPAL patients have a similar or better OS compared to AML patients, but not ALL patients [3,7]. Immunophenotype has also been shown to influence prognosis, with T/myeloid patients having a decreased CR rate and OS compared to those with B/myeloid MPAL [8].

To date, there have been no prospective trials to help guide treatment for patients with MPAL, but retrospective studies have shown that treatment with ALL-directed chemotherapy regimens followed by allogeneic stem-cell transplant (ASCT) offers the best outcome for Philadelphia-chromosome negative MPAL patients [9]. This leaves a clinical dilemma for patients who fail initial treatment guidelines as there is no clear evidence for how to proceed. This case report describes a patient with relapsed B/myeloid MPAL who achieved complete remission with incomplete count recovery (CRi) after treatment with a novel combination of azacitidine, a hypomethylating agent, and the FMS-like tyrosine kinase 3 (FLT3) inhibitor gilteritinib, without proceeding ASCT.

This article was previously presented as an abstract with the title “Mixed Phenotype Acute Leukemia: A review of a rare condition,” at the Society of Hospital Medicine Converge meeting in May 2021.

## Case presentation

**Figure 1:**
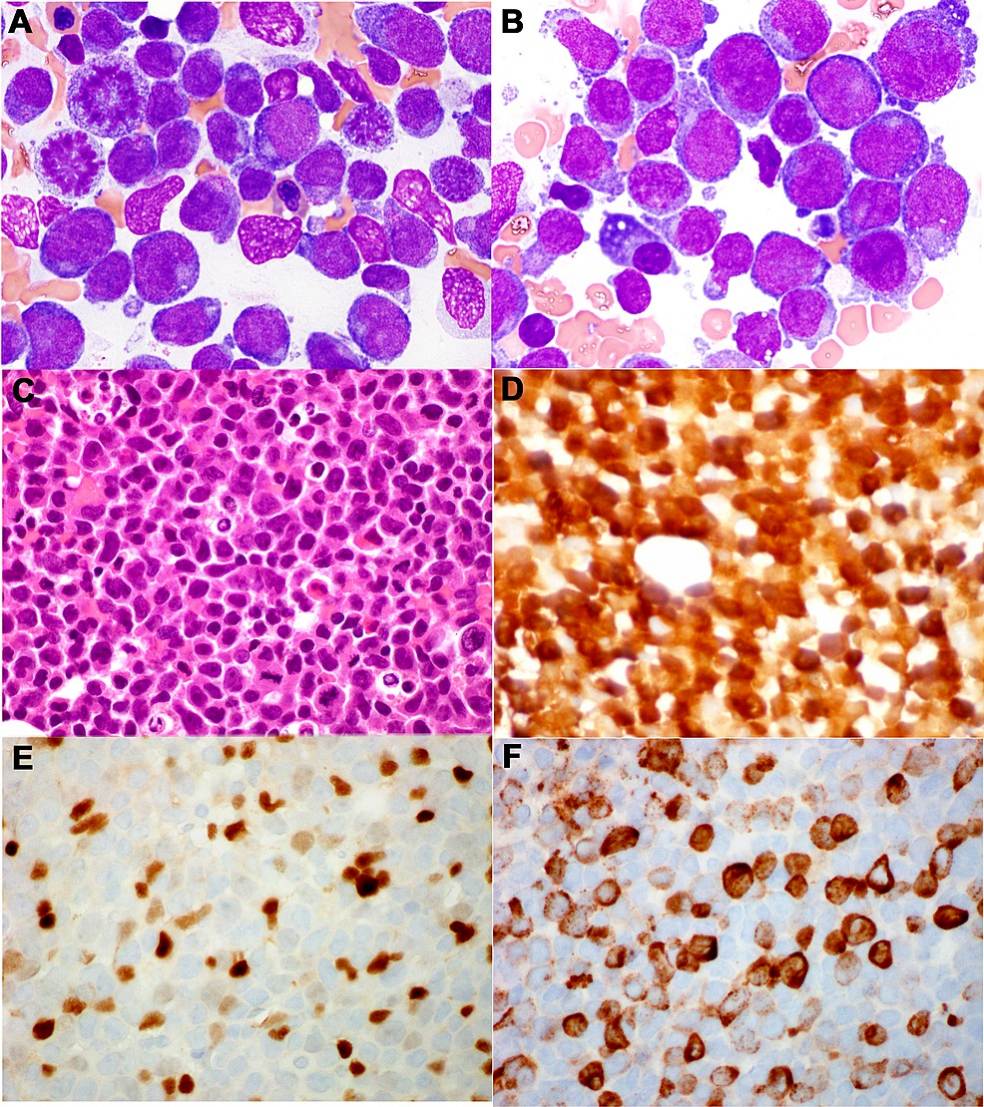
Bone marrow biopsy with B/myeloid mixed phenotype acute leukemia. A-B) the bone marrow aspirate smears from the patient diagnosed with mixed phenotypic leukemia, B-myeloid, demonstrated the two distinct populations of blasts: 1) small in size with high N: C ratio, round to oval nucleoli, inconspicuous nucleoli with scant or abundant cytoplasm, a subset of which displayed hand-mirror-like morphology; 2) large, smooth to irregular nuclear contour, deeply basophilic cytoplasm, and some with prominent nucleoli, (Wright-Giemsa, ×1000). C) The bone marrow core biopsy showed sheets of immature precursors/blasts, variable in size, with mild to moderate nuclear irregularity and brisk mitoses (H&E ×600). D-F) These blasts were positive for lysozyme (D), PAX5 (E, scattered), and myeloperoxidase (F, subset) (immunoperoxidase, ×200).

A 55-year-old woman with controlled hypertension and no other significant medical comorbidities presented to an outside hospital with nausea, generalized weakness, progressive dyspnea, and fatigue following treatment for sinusitis. Initial blood counts revealed a white blood cell count of 135 × 10^9^/L with anemia and thrombocytopenia. She received three rounds of leukapheresis for concern of hyperviscosity and was treated with hydroxyurea for cytoreduction. The patient was transferred to our cancer center for further management. Repeat blood counts showed a white blood cell count of 39.8 × 10^9^/L with 80% blasts, and a post-transfusion hemoglobin level of 8.3 g/dL and platelet count of 50 × 10^9^/L. Bone marrow biopsy showed hypercellular bone marrow effaced by sheets of blasts comprising approximately 80% of total cellularity (Figure 1) and the concurrent flow cytometric analysis of the bone marrow aspirate revealed two populations of CD34+ blasts. The first population consisted of B-lymphoblasts with strong expression of surface CD19, cytoplasmic CD79a, and intranuclear terminal deoxynucleotidyl transferase (TdT), along with aberrant expression of some myeloid antigens (CD13 and CD33). Immunohistochemistry (IHC) showed positivity for paired-box domain 5 (PAX5), a B-cell-specific transcription factor. The second population consisted of myeloblasts (with CD13, CD33, CD117, and HLA-DR). They lacked CD19 but showed cytoplasmic CD79a, and IHC showed positivity for myeloperoxidase and lysozyme (Figure 1). The patient was diagnosed with B/myeloid MPAL with bilineal blasts.

Upon further characterization of the malignancy, the cytogenetic analysis showed 47,XX,+6(3)/46,XX(17) and fluorescence in-situ hybridization (FISH) was negative for the Philadelphia chromosome, KMT2A, and chromosomal arrangements seen in myelodysplastic syndrome, including del(5q), -5, del(7q), -7, +8, del(20q), and del(17p). Polymerase chain reaction (PCR) showed a BCR-ABL1 p190 ratio of 0.000002% and was undetectable for p210. Next-generation sequencing (NGS) revealed mutations in runt-related transcription factor 1 (RUNX1) (VAF of 47.53%) and Wilms’ tumor protein 1 (WT1) (VAF of 17%). FLT3 PCR was positive for an internal tandem duplication (ITD) mutation (VAF of 25.7%) with a second mutant allele (VAF of 12.5%) but was negative for a tyrosine kinase domain (TKD) mutation. The clonoSEQ® Assay (Adaptive Biotechnologies, Seattle, Washington, USA) showed two dominant IgH sequences.

The patient received induction chemotherapy with hyper-cyclophosphamide, vincristine, doxorubicin (also known by its trade name, Adriamycin), and dexamethasone (CVAD) and midostaurin. Cerebrospinal fluid analysis revealed a small number of atypical cells, so the patient was given intrathecal methotrexate and cytarabine, with no recurrence of central nervous system (CNS) malignancy to date. Her induction course was complicated by neutropenic fevers, acute otitis media, herpes simplex virus-positive lip lesions, a right internal jugular vein thrombosis secondary to central line placement, and ulceration over the anterior border of the right mandibular ramus. Hyper-CVAD Arm 3A was complicated by both a non-ST-segment elevation myocardial infarction (NSTEMI) and a right middle cerebral artery stroke, which delayed her progression to ASCT (Table 1). During this time, blood counts showed blasts up to 11%, and a bone marrow biopsy revealed a hypercellular marrow with diffuse involvement of myeloid-predominant MPAL (67%) with less than 5% B-lymphoblasts. She was treated with hydroxyurea for cytoreduction and then began treatment with the second-generation FLT3 inhibitor gilteritinib.

**Table 1:** Summary of treatment response and related complications. CR: complete response; PD: progressive disease; RD: residual disease; CRi: complete response with incomplete count recovery; CVAD: cyclophosphamide, vincristine, doxorubicin (also known by its trade name, Adriamycin), and dexamethasone

Cycle	Systemic Treatment	Response	Toxicities and Complications
1	Hyper-CVAD + Midostaurin	CR	Anemia, thrombocytopenia, neutropenic fevers, right internal jugular vein thrombosis, acute otitis media, ulcer overlying right mandibular ramus, herpes simplex virus-positive lip lesions
2-3	Hyper-CVAD	PD	Anemia, thrombocytopenia, non-ST segment elevation myocardial infarction, influenza A, right middle cerebral artery stroke, persistent epistaxis
4	Gilteritinib	RD	Anemia, thrombocytopenia
5-12	Gilteritinib + Azacitidine	CRi	Anemia, thrombocytopenia, diarrhea

After four months of treatment with gilteritinib, bone marrow biopsy showed a reduction in blasts, but residual disease with 25% hypocellular marrow and 15.2% myeloblasts. She continued treatment with gilteritinib and began receiving concurrent azacitidine. After two cycles of combination therapy, a follow-up bone marrow biopsy was 10-15% cellular with 3% blasts, with the overall clinical findings consistent with CRi. The patient continues to receive treatment with both azacitidine and gilteritinib as she is currently not a candidate for ASCT due to a poor Karnofsky performance score as a result of her prior stroke. The patient remains platelet transfusion-dependent, but she has maintained CRi for eight months with this treatment.

## Discussion

Given the rarity of MPAL, there is limited prospective data on treatment management algorithms in patients failing first-line therapy. This patient was initially treated in the traditional approach with hyper-CVAD, however, due to complications with myocardial infarction and cerebral vascular accident, she was unable to complete the standard of care with an ASCT, and, unfortunately, relapsed. The patient’s bone marrow biopsy at relapse showed a majority of myeloid cells with an FLT3 ITD mutation.

FLT3 is a receptor tyrosine kinase (RTK) found in hematopoietic cells that is involved in regulating cell survival, proliferation, and differentiation [10]. FLT3 mutations are found in approximately 30% of cases of AML and result in constitutive activation of the RTK, promoting survival of the leukemic cells [11]. Mutations in the internal tandem domain of FLT3 confer a poor prognosis with reduced OS and disease-free survival, and higher rates of relapse [12]. Additionally, AML patients with FLT3 ITD mutations were found to have significantly reduced survival after relapse [13]. Given our patient’s failure to midostaurin as the first-line therapy, other choices are considered. Gilteritinib is a second-generation FLT3 tyrosine kinase inhibitor that has been shown to significantly increase the survival of relapsed or refractory FLT3-mutated AML patients, compared to chemotherapy [14]. Treatment of this patient’s relapsed MPAL with gilteritinib alone resulted in regression of bone marrow blasts but did not induce remission. Azacitidine, a DNA hypomethylating agent, was added in combination with gilteritinib to enhance therapeutic efficacy. After two cycles of therapy, the patient achieved CRi and has maintained CRi for eight months with continued treatment.

Additionally, due to the patient’s declined performance status, cytotoxic chemotherapy was no longer an option. The management of this patient with a combination of azacitidine and gilteritinib provides an example of a successful treatment regimen when other treatment avenues are not feasible. MPAL potentiates a poor prognosis at baseline and the prognosis worsens with relapsed or refractory disease. In one study, the relapse rate of MPAL was higher compared to AML, and the CR rate of MPAL after relapse was comparatively lower. Additionally, the OS and disease-free survival of MPAL were also significantly lower compared to both AML and ALL [6]. Using a combination regimen with targeted therapy illustrated its utility in rare and difficult to treat malignancies. Although this treatment regimen lacks curative potential, and the patient’s remission may evaporate with time, the combination approach gave her more time and a greater quality of life. While, to our knowledge, this is the first case illustrating this combination therapy in MPAL, other case reports document other combination approaches in treating this disease.

A review of the available literature yielded two case reports of interest that demonstrate success in treating B/myeloid MPAL patients with targeted approaches. In one study, two treatment-naïve patients were treated with a combination of a hypomethylating agent and the B-cell lymphoma-2 (BCL-2) inhibitor, venetoclax. Both patients subsequently received ASCT after achieving CR [15]. Additionally, blinatumomab, a bi-specific T-cell engager (BiTE) monoclonal antibody directed against CD19 was used to treat a patient whose performance status declined while being treated with hyper-CVAD. The patient achieved minimal residual disease (MRD)-negative morphologic and cytologic remission after three rounds of blinatumomab and then proceeded to ASCT [16]. Overall, however, there is currently very little published research documenting efforts to treat MPAL with targeted therapies.

Although this case report demonstrates an exciting response to two medications that are not front-line therapies, or even within the treatment algorithm, a single case cannot fully validate this regimen. A single case is limited by a lack of statistical power and is confounded by many patient factors. Medications behave differently in each individual. Azacitidine and gilteritinib are no exception. These findings represent this patient’s response to these drugs which could result from a less aggressive disease or perhaps an MPAL that is more sensitive to these medications than seen in other patients. Nonetheless, this case provides an example of a treatment that could be considered in a similar situation, especially when there are no other suitable alternatives.

MPAL is an uncommon form of hematologic malignancy and therefore has limited data on regimens to use after front-line therapy. This provides multiple avenues for future research. Prospective data would be beneficial to establish an algorithmic approach to salvage therapy due to the high risk and poor prognosis associated with MPAL and the lack of treatment options. Therefore, novel therapeutic approaches for MPAL should be investigated in order to prolong patient survival and improve quality of life.

## Conclusions

Current guidelines for the treatment of MPAL should be improved, especially for those for whom ASCT is not an option. Here, we described a patient with relapsed FLT3-mutant MPAL who had a remarkable response to salvage therapy with azacitidine and gilteritinib and has remained in remission for eight months. We chose to document this case in order to highlight a unique treatment regimen that can be used when there is no alternative, as well as to emphasize the need for more quality research, particularly prospective studies, to help improve treatment guidelines for MPAL. Targeted therapy, such as with gilteritinib, could be an excellent candidate for salvage therapy in cases of MPAL with targetable somatic mutations.
